# Chloroform

**Published:** 1848-03

**Authors:** 


### THE WESTERN JOURNAL.

THIRD SERIES.—VOL. I.—No III.

LOUISVILLE, MARCH 1, 1848.

### CHLOROFORM.

Although we have already devoted so much of our present Number to the subject of chloroform, we propose giving in this place a few additional details on the same subject, persuaded that our readers cannot help participating in the deep interest so generally felt in this new anæsthetic agent. If the profession in America were slower than their brethren in Europe to adopt ethereal inhalation, we believe the same thing cannot be said of their disposition to test the virtues of chloroform. The cost of the article, which in London and Paris was so great that it was feared that it must seriously limit its application, is already reduced to a point in this country which places it within the reach of all, and favors its indiscriminate use by unprofessional as well as professional persons. The result of this will be, that we shall learn in the shortest possible period the capacity of the agent for mischief as well as for good. When first brought into notice, by Prof. Simpson, chloroform sold in London at four shillings, and in Paris at seventeen francs, an ounce; the same quantity is now sold in Louisville at less than thirty cents.

*Physiological Action of Chloroform.*—We have repeatedly inhaled the protoxide of nitrogen and the vapor of chloroform, and our first sensations under the influence of the two were identical; they were of the most delightful character, and we found it difficult to resist the propensity to repeat the inhalations again and again. After a few inspirations of the chloroform, we lost our consciousness, and fell into a pleasant sleep, a state which we have never succeeded in inducing by the exhilarating gas. From the experiments made by a committee of the Medico-Chirurgical Society of Edinburgh, the physiological action of chloroform would appear to be precisely that of ether. They produce loss, 1st, of the cerebral functions; 2d, of the spinal functions; and 3dly, of those of the medulla oblongata. A smaller quantity of chloroform is required to produce the desired effect, and the unconsciousness is obtained by it more rapidly than by the use of ether; but whether the effects are more or less persistent, is a question not yet decided among experimentalists. Roux believes they are less so, but Velpeau thinks the insensibility is more prolonged. Longer experience and more trials on subjects of every variety of temperament, are required to settle this point. We have remarked that the exhilarating effects are intermitting, the subject, after a moment or two of inactivity, growing hilarious and noisy, again becoming lethargic, to start up soon again, and sing, dance, recite, laugh, or cry, with as much vivacity as ever. Amussat has found in animals and in man that chloroform, like ether, causes the arterial blood to assume a dark color, and to resemble closely venous blood. This condition of the circulating fluid is more apt to be produced when the vapor is inhaled from an apparatus which limits the supply of atmospherical air. When breathed from a sponge, or a handkerchief, the blood is less affected, and the subject is in no danger of falling into a state of asphyxia. For this reason all instruments for administering these vapors are now generally rejected, and the simpler appliance of the sponge or handkerchief has taken their place.

The advantages of chloroform over ethereal inhalation are so decided, that the former must soon supersede the latter in general practice. Not only are the effects induced by a smaller quantity, and in a shorter time, but its vapor is pleasanter to the taste, and less irritating to the lungs; and when sensibility is restored, it is without the headache and nausea which so often follow the administration of ether. We are among those who regard the letheon as a precious boon to the human race, and there are cases in which it will probably continue to be employed both by the surgeon and physician, but its great merit, we must believe, consists in having led to the discovery of the anæsthetic virtues of chloroform. Physicians have used ether inhalation in a great variety of diseases, and the testimony of some of the most eminent among them is, that it is applicable in every form of pain incident to the human frame. In all spasmodic diseases especially, its effects are most grateful. The experience of physicians with chloroform is necessarily still very limited, but enough has been shown to justify the most extensive application of it to diseases marked by acute suffering.

Dr. Simpson, the distinguished Professor of Midwifery in the University of Edinburgh, was one of the first to apply ether inhalation in parturient women, and has been the most zealous advocate of the practice. In a late publication on the subject of chloroform in obstetric practice, he concludes the history of several interesting cases, in which insensibility was induced by this agent, with the following remarks:

"The preceding instances afford, perhaps, a sufficient number of examples of the use of chloroform in natural labor. In these and in all others which I have seen, or that have been reported to me, the immediate effects of the chloroform have been delightful. The mothers, instead of crying and suffering under the strong agonies and throes of labor, have lain in a state of quiet, placid slumber, made more or less deep at the will of the medical attendant, and, if disturbed at all disturbed only unconsciously from time to time by the recurring uterine contractions producing some reflex or automatic movements on the part of the patient—like those of a person moving under any irritation of the surface, or from the touch of another, though still in a state of deep sleep. Nor have the ultimate consequences and results been less happy. No difficulties have been met with in the third stage; and the uterus has contracted perfectly after delivery. I never saw mothers recover more satisfactorily or rapidly,—or children that looked more viable. And the practice is not a great blessing to the patient merely, it is a great boon also to the practitioner. For whilst it relieves the former from the dread and endurance of agony and pain, it both relieves the latter from the disagreeable necessity of witnessing such agony and pain in a fellow-creature, and imparts to him the proud power of being able to cancel and remove pangs and torture that would otherwise be inevitable. It transforms a work of physical anguish into one of painless muscular effort; and changes into a scene of sleep and comparative repose, that anxious hour of female existence, which has ever been proverbially cited as the hour of the greatest mortal suffering."

But, he adds, "the effects of the superinduction of anæsthesia in parturition are, if possible, still more marked and beneficial in cases of morbid labor and operative delivery;" in illustration of which he gives the history of several cases of turning, of the application of the forceps, and of embryulsio.

In an appendix to this pamphlet of Dr. Simpson's, which has been republished at Boston, Dr. Warren's more extended experience with chloroform is given. He says:—"The effects of the new anæsthetic I have had opportunity of observing in a considerable number of operations, and among these anchylosis, strangulated hernia, amputation, removal of tumors, etc. It was a great satisfaction to me to do an operation for strangulated hernia—an operation which I have so often seen attended with severe distress—on a gentleman with a bad strangulation, who, during the ten minutes required, lay as perfectly tranquil as a subject on the dissecting table of the anatomist, and who directly after its completion awoke in his right mind, and with no knowledge of the operation. This gentleman accidentally swallowed about half a teaspoonful of pure chloroform without any material ill consequence."

Dr. Channing has a letter in this appendix in which he states, that he is about to put to press a volume on the employment of etherization in childbirth, and adds that he has witnessed the happiest effects from chloroform "in all cases in which he has employed it."

Dr. C. T. Jackson, whose name is connected with the discovery of the letheon, bears the same testimony to the virtues of chloroform, and adds the following remarks as to a more diversified application of the agent:

"I have administered both chloroform and sulphuric ether vapors to the insane with very agreeable effects, the patients falling into a deep sleep and gaining, as it were, a short respite from their distressing delusions which had haunted them continually and prevented sleep. This period of rest was found to be quite salutary, and it is probable that critical changes in those troubles may be induced by this new method of treatment. In one case, that of a furious maniac, who had torn his clothes entirely from his body, and whom we took from his cell and treated with sulphuric ether vapor, the relief by inhalation of the ether was most remarkable. This patient who had not slept for several nights, was at once subdued by this treatment and put into a deep sleep of insensibility and laid on his bed where he remained asleep all the afternoon and evening of that day, and became quite tranquil when he awoke and has up to the latest accounts not suffered any new paroxysm. In the hands of skilful Physicians, for the insane these remedial agents may prove a great blessing.

"I have long since urged the application of etherization in cases of intermittent fever, but have not yet seen any account of its trial. I would now renew my invitation to physicians in the south and west to try both chloroform and ether in that troublesome disorder. If the chloroform will break the chills and bring on, as it probably will, a copious perspiration (one of its common effects) it will prove a great blessing, and the remedy will be found quite portable, since only thirty or forty drops of this agent is required to produce its proper effects."

Professor Gross has used the chloroform successfully in several surgical operations, and Professor Miller has employed it with the happiest results in a case of difficult labor. But we will not take leave of the subject without adding, that a woman is reported to have died in the hands of a Dentist, in Cincinnati, from the effects of this agent.

It is important to employ a genuine article, since the agreeable action depends upon its purity. A test, easy of application, is said to consist in dropping the chloroform into water, mixed with an equal quantity of sulphuric acid. When perfectly pure it retains its transparency as it sinks into the water, but when otherwise it becomes cloudy.

